# Early Nutrition with Different Diets Composition versus Fasting on Immunity-Related Gene Expression and Histomorphology of Digestive and Lymphoid Organs of Layer-Type Chicks

**DOI:** 10.3390/ani11061568

**Published:** 2021-05-27

**Authors:** Shaimaa Selim, Nazema S. Abdel-Megeid, Manal K. Abou-Elnaga, Samy F. Mahmoud

**Affiliations:** 1 Department of Nutrition and Clinical Nutrition, Faculty of Veterinary Medicine, University of Menoufia, Shibin El-Kom 32514, Egypt; 2 Department of Cytology and Histology, Faculty of Veterinary Medicine, University of Sadat City, Sadat City 32897, Egypt; nazemah.abdelmageed@vet.usc.edu.eg; 3 Department of Poultry and Fish Production, Faculty of Agriculture, University of Menoufia, Shibin El-Kom 32514, Egypt; manal.abouelnaga2@agri.menofia.edu.eg; 4 Department of Biotechnology, College of Science, Taif University, P.O. Box 11099, Taif 21944, Saudi Arabia; s.farouk@tu.edu.sa

**Keywords:** early feeding, histology, immunity, gene expression, liver, proventriculus, chicks

## Abstract

**Simple Summary:**

With the continuous improvement in the progress of poultry industry, a better understanding of the avian immune system is necessary. A prolonged holding period (36–72 h), along with a delay in access to feed and/or water post-hatching, has a negative influence on performance, intestinal histomorphology, and the immune system development of chicks. Therefore, the present study aimed to investigate the effect of early feeding with different diet composition or delayed feeding during the first 72 h of chick’s life on the expression of immunity-related genes and histomorphology of digestive and lymphoid organs of layer-type chicks. Early nutrition post-hatching had no negative effect on the development of the lymphoid and digestive organs in chicks. Histomorphological examination revealed an increase in cortex and cortex:medulla of thymus and bursa in the early fed groups compared to the fasted ones, with resultant impacts on the primary lymphoid organs. Higher germinal center areas and white pulp of the spleen were recorded in the early fed chicks, implying augmented proliferation and maturation of B cells in the secondary lymphoid organs. In the liver, a strong positive reaction to Best’s carmine stain in the early fed groups, indicating that the liver of these chicks had numerous glycogen granules in the cytoplasm of hepatocytes. The expression levels of splenic-immunity related genes were up-regulated in most of the early fed chicks at 14 day of age. Our findings suggested that early feeding post-hatch can modify the splenic-immunity related genes and modulate the histomorphology of the digestive (liver and proventriculus) and lymphoid organ in layer-type chicks during the early life post-hatching.

**Abstract:**

Early feeding post-hatching (EFPH) can impact the immune response and modify the immunity-related gene expression. Therefore, we aimed to examine the effects of EFPH with different diets composition versus fasting during the first 72 h of chick’s life on the histomorphological structures of the liver, proventriculus, central and peripheral lymphoid organs, and immunity-related genes in layer-type chicks during the brooding period. A total of 400 chicks were randomly allotted into 4 groups with 4 replicates each. The experimental groups during the first 72 h of life were: feed and water deprivation (control, T1), feeding a starter layer diet (20% CP and 11.84 MJ/kg ME, T2), feeding a starter layer diet contained 3% molasses in its composition (20% CP and 11.81 MJ/kg ME; T3), and feeding a starter broiler diet (23% CP and 12.68 MJ/kg ME, T4). After the first 72 h of chick’s life, all chicks were fed ad libitum the T2 diet. EFPH had no negative effect on the development of the lymphoid or digestive organs in chicks. Greater relative weights of the spleen and bursa of Fabricius (*p* < 0.05) were observed in the early fed chicks compared to control at day 14 of age. Histomorphological examination revealed an increase (*p* < 0.05) in thymus cortex and cortex:medulla in the T3 and T4 groups compared to the fasted ones at day 28 of age. Pelicae height, follicular width, cortex, and cortex:medulla of bursa were improved (*p* < 0.01) in the fed groups compared to fasted chicks, with resultant influences on the primary lymphoid organs. Compared to control, higher germinal center areas and white pulp of the spleen (*p* < 0.05) were recorded in the early fed chicks, implying augmented proliferation and maturation of B cells in the secondary lymphoid organs. In the liver, a strong positive reaction to Best’s carmine stain in the early fed groups, indicating that the liver of these chicks had numerous glycogen granules or greater glycogen density in the cytoplasm of hepatocytes. There was a significant enhancement in the proventriculus mucosal and gland thickness, as well as fold height (*p* < 0.05) in the early fed chicks. The expression levels of splenic Toll-like receptor 2, interleukin 4, tumor necrosis factor α, and interferon gamma were up-regulated (*p* < 0.01) in most of the early fed chicks (T2, T3, and T4) compared to fasted ones at 14 day of age. In conclusion, EFPH could modify the splenic-immunity related genes and modulate the histomorphology of the digestive (liver and proventriculus) and lymphoid organs in layer-type chicks during the brooding period.

## 1. Introduction

During the last two decades, a significant enhancement has been made on the functional characteristics of layer- and meat-type poultry through genetic selection; accordingly rising the prevalence of early chick mortality, possibly due to immunosuppression and a reduced resistance to infections. With the continuous improvement in the progress of poultry industry, a better understanding of the avian immune system is necessary. The immune system development of the newly hatched chicks is influenced by numerous factors, one of the most critical factors is the early feeding post-hatch (EFPH) [1,2,3,4]. Newly hatched chicks are commonly pulled out when the majority of them have hatched, causing chicks to be deprived of feed or water for 24–72 h. In addition, other common management practices such as sex determination, counting, vaccination, and transportation are accountable for the delayed feeding post-hatch (PH) [4,5].

Prolonged holding period (36–72 h) along with a delay in access to feed or water have negative influences on chicks because of dehydration and energy depletion [4,6]. Feed deprivation (FD) PH could adversely impact the growth performance, intestinal histomorphology, the rate of nutrient absorption, and the immune response, both in broiler- and layer-type chicks [3,4,7,8,9,10]. Metabolic changes caused by early FD could possibly diminish the lymphoid organs growth, and further increase the susceptibility to diseases and compromise the immune response [2,4,6], resulting in a greater production cost of birds. The intestinal development, in particular villi length and number of enterocytes, as well as the lymphoid organs growth occur more rapidly after the first 7 days PH with intensive early development up to 21–35 days PH in broilers [11,12] and between 14 and 42 days PH in layers [9], accordingly, chicks during the first week PH have an insufficient immune response due to the insufficiency of the immune systems development [1,4,9]. The immune system of newly hatched birds (broilers and layers), in particular the mucosal immune system, necessitates feed intake for prompt development and maturation [1,6,9,10]. In layers, Simon et al. [9] reported that early-fed chicks had higher live weight from day 3 through day 35 PH, greater relative weight of spleen (until day 49 PH) and bursa of Fabricius (day 6 and day 9 PH), and an earlier onset of ileal IgA expression (day 9 vs. day 14 PH) than FD chicks. The application of nutritional strategies can efficiently stimulate the early development and maturity of the immune system and boost the innate immunity of chicks [9,11].

The yolk sac of the hatchlings has high protein and fat contents but little carbohydrate content [13]. Due to the fat not playing a part in the metabolic synthesis of glucose, gluconeogenesis is mostly originated from body reserves and yolk sac protein [14]. Newly hatched chicks have low efficiency in utilizing nutrients, and the inclusion of highly digestible energy sources such as simple glucose, sucrose, or glucose-based materials in their early diets is believed to improve the performance of chicks [5]. These materials are considered as a good source of glucose and are efficiently utilized by newly-hatched chicks for their abrupt glucose demands PH [14]. Moreover, partial substitution of starch with simple sugars (4–8%) in pre-starter diets potentially enhanced the chick’s performance and can be applied to mitigate the adverse effects of delayed access to feed PH [15]. Sugarcane molasses is cheap, easily available in Egypt, and contain approximately 56% total sugars (sucrose, fructose, and glucose). Molasses can be serving as a source of energy in the poultry feeds and was used to improve the palatability and feed intake of chicks with a dietary recommended level not more than 5–7% in the mash chicks’ diets [16]. On the other hand, it was documented that increasing dietary protein (24.6% vs. 21.4%) and digestible amino acids (114% vs. 100% of cobb recommendation) in the pre-starter diets (day 1 to day 10 of age) greatly enhanced the growth performance with significant changes in the intestinal weight and histomorphology of broilers [17]. Early nutrition on easily digestible carbohydrates, protein or amino acids either in ovo or in the chicks’ early diets were applied to decrease the demand for gluconeogenesis, meet the needs for rapid growth, enhance the intestinal development, and augment the immune response during the early PH period [3,5,7,17,18,19,20].

EFPH of chicks is a stimulus to the development and functioning of the immune system, the primary and secondary immune organs, during the early stages of life [2,4,8] and the health status of chicks on long-term [20]. After activation, the naive T cells is differentiated either into T helper 1 (Th1) cells (cellular immunity and pro-inflammatory function), or Th2 effector cells release cytokines that enhance humoral immunity [21]. The development of these cells begins during the embryogenic stage and continues PH. Recently, it has been reported by Song et al. [22] that the lowest point of the immunity in broiler chickens appeared within day 6 to 13 of age, whereas the highest levels of the immune status occurred within 30 and 34 days of age, and the key indicators of this pattern were blood and splenic cytokines. In layer-type chickens, the intestinal cytokine expression levels were enhanced between day 14 and 42 PH compared to day 3–4 PH [9,23]. In both breeds (broiler and layer chicks), EFPH has been recorded to increase the weights of lymphoid organs and enhanced the immune response PH when compared with the FD chicks [1,4,6,8,9,18]. Furthermore, the impacts of feed and water access versus fasting in layer-type chicks are mostly studied [9,10], ignoring the effects of EFPH or FD on the histomorphology of digestive (liver and gizzard) and lymphoid organs as well as the effect of diet composition [9,10].

Very few studies have been performed to assess the effect of early feeding or feed and water deprivation PH (FWDPH) on the growth performance and intestinal morphology in both broiler and layer-type chicks but to our knowledge little information is existing in the literature about the effect of EFPH with different diets composition versus FWDPH on the expression of immunity-related genes and histomorphology of digestive (proventriculus and the liver) and lymphoid organs (bursa of Fabricius, spleen, and thymus gland) of layer-type chicks. Therefore, the present study aimed to determine the effect of EFPH with different diet composition or DF during the first 72 h of chick’s life on the expression of immunity-related genes and histomorphology of digestive and lymphoid organs during the brooding period of layer-type chicks. The hypothesis of the present study was that delayed feeding PH will influence the early development of digestive and lymphoid organs and alter the expression of immunity-related genes of chicks.

## 2. Materials and Methods

### 2.1. Ethical Approval

The procedures adopted in this trial were performed according to the guidelines as approved by the Animal Ethics Committee of the Faculty of Agriculture, University of Menoufia, Menoufia, Egypt (No. 4/2017).

### 2.2. Hatching Eggs, Experimental Treatments and Management

The experimental design was previously published in Abou-Elnaga and Selim [7]. Eggs from Norfa layer flock (White Leghorn × Fayoumi × White Baladi) weighing between 40 and 45 g were collected and incubated in a hatchery (Poultry Research Farm, Faculty of Agriculture, Menoufia University, Menoufia, Egypt). On day 18 of incubation, eggs were moved to the hatchery. A group of 400 hatched chicks (200 males + 200 females) was used in the current trial. Hatching trays were divided with partitions and 4 different treatments were applied using a completely randomized design. Each treatment had 4 replicates with 25 chicks per replicate. Treatments were fasting (control, T1; no feed or water), feeding a layer starter diet (20% CP and 11.84 MJ/kg ME; T2), feeding a starter diet containing 3% molasses (20% CP and 11.81 MJ/kg ME; T3), and feeding a starter broiler diet (23% CP and 12.68 MJ/kg ME, T4) during the first 72 h post-hatch. Immediately, all chicks after fasting or feeding the experimental diets for the first 72 h of their life were fed layer starter mash diet (T2) *ad libitum* for a period of 42 days. All chicks were moved from the hatchery after 72 h PH and kept in the floor pens. The ingredients and the proximate chemical analysis of the diets used in the current trial were published previously in Abou-Elnaga and Selim [7]. All chicks were reared under the same standard management practices and routine vaccination during the experimental period.

### 2.3. Lymphoid and Digestive Organs Histomorphology

At 14 and 28 days PH, 8 chicks (both the sex) from each group (2 per replicate), within the average live weight, at each time point were killed by cervical dislocation. The weight of liver [7], proventriculus, bursa, spleen, and thymus were recorded and expressed as per cent of live weight. The routine histological techniques were performed according to Bancroft and Stevens [24]. Tissue samples were rapidly placed in 10% neutral buffered formalin for approximately 48 h, then tissue processing occurred firstly by transferring samples to the dehydration process through ascending grades of alcohols from 70% to 100%, following by the clearing process through passing in a clearing agent as methyl benzoate then embedding in paraffin wax at melting point 56 °C in a hot air oven. The paraffin tissue blocks were cut by using a rotatory microtome. All samples were sectioned at 4–6-μm thickness. Sections were mounted on a clean glass slides and stained with Hematoxylin and Eosin (H and E) for the general histological examination and Best’s carmine stain for detection the distribution of glycogen in the liver tissue [24]. After glass slides being dried, cross sections from tissue samples (proventriculus, liver, bursa of Fabricius, thymus, and spleen) were examined using a light microscope. The photomicrographs from the selected specimens were taken using Leica digital camera connected with bilocular microscope for better illustration of the findings. The Morphometric measurements were performed according to Madej et al. [12], Sikandar et al. [25], and Sayrafi and Aghagolzadeh [26]. In proventriculus, height of the proventriculus fold, thickness of mucosa, diameter of compound gland, and Pelicae height were determined. In bursa of Fabricius, follicular width and thickness of follicular cortex and medulla were calculated. In thymus, thickness of cortex and medulla were measured. In spleen, the thickness of area of white pulp and red pulp were performed. Splenic germinal center areas were measured as described by Madej et al. [12] and Sikandar et al. [25]. To estimate the hepatic glycogen density in the cytoplasm, a scale was performed ranging from 1 (low glycogen density) to 4 (very high glycogen density), depending on the staining of glycogen granules with the Best’s carmine stain. Four histologists (masked to the groups) issued the scores.

### 2.4. Immunity-Related Gene Expression

Eight chicks from each treatment group at each time point (2 chicks per replicate) were selected and killed with cervical dislocation for the gene expression analysis. Splenic samples were dissected carefully and frozen at −80 °C until further analysis. Splenic immunity was performed by a quantitative measurement of the mRNA expression of interleukin 4 (IL4), interferon gamma (IFNγ), tumor necrosis factor α (TNFα), and Toll-like receptor 2 (TLR2) on day 14 and 28 of age. The total RNA extraction, preparation, and cycling conditions for real-time polymerase chain reaction (PCR) were determined as described previously by Bhanja et al. [3]. The oligonucleotide sequences of the primers used in the current study were listed in Table 1. Amplification curves and cycle threshold (CT) values were measured using Stratagene MX3005P software (Agilent Technologies, Inc., Santa Clara, CA, USA). The reference housekeeping gene was 28S rRNA. Relative mRNA expression was calculated using the 2^(−ΔΔCt)^ method, and the results were recorded as a fold change [27].

### 2.5. Statistical Analysis

Normal distribution of the data was determined with the Kolmogorov–Smirnov test. The obtained data from the effect of early nutrition with different diets and fasting on the digestive and lymphoid organs histomorphology and splenic gene expression at two time points were subjected to One-way Analysis of Variance using GLM procedures of SPSS software (SPSS, version 21, SPSS Inc., Chicago, IL, USA). Statistically different means were separated using Duncan’s multiple range test at *p* < 0.05.

## 3. Results

### 3.1. Histomorphology of Lymphoid Organs

#### 3.1.1. Thymus Gland

**Figure 1:**
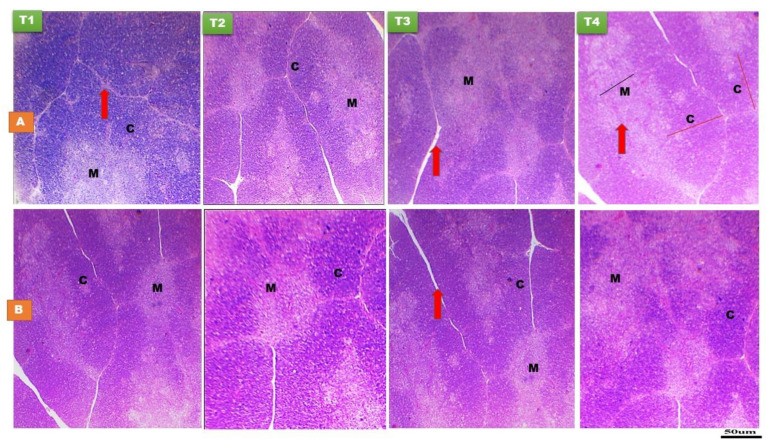
Histological structure of thymus gland at day 14 (**A**) and day 28 (**B**) of age of layer-type chicks exposed to fasting (control, T1) or early feeding on a layer starter diet (T2), a layer starter diet contained 3% molasses (T3), and a broiler starter diet (T4) during the first 72 h post-hatching. M, medulla; C, cortex. Thymus trabeculae (red arrow). Area of cortex with dark staining (red line) and area of medulla with light staining (black line) (H and E ×40 and 100).

There was non-significant (*p* > 0.05) effect of early nutrition PH on the relative weight of thymus or its medulla thickness, both at day 14 and day 28 of age (Figure 1 and Table 2 and Table 3). The histomorphological structure of the thymus gland was normal in all treatments and at both time points (Figure 1). Thymus gland is composed of incompletely separated lobules. Each thymic lobule is divided into an outer dark staining cortex and an inner light staining medulla due to the increased numbers of T-lymphocytes and other cortical cells than that in medulla. Intensive growth of thymic lobules was detected up to day 28 PH (Figure 1). A significant effect of early nutrition PH (*p* < 0.05) was noticed for the cortex of thymus, indicated that the T3 group at day 14 of age and the T2, T3, and T4 groups at day 28 of age had the greatest cortex thickness compared to the fasted ones. The cortex to medulla ratio was higher (*p* < 0.05) in the T3 and T4 groups at day 28 of age than the fasted and T2 groups (Figure 1 and Table 3). However, there was a non-significant difference in the cortex to medulla ratio among the treatment groups at day 14 of age (Table 2).

#### 3.1.2. Bursa of Fabricius

**Figure 2:**
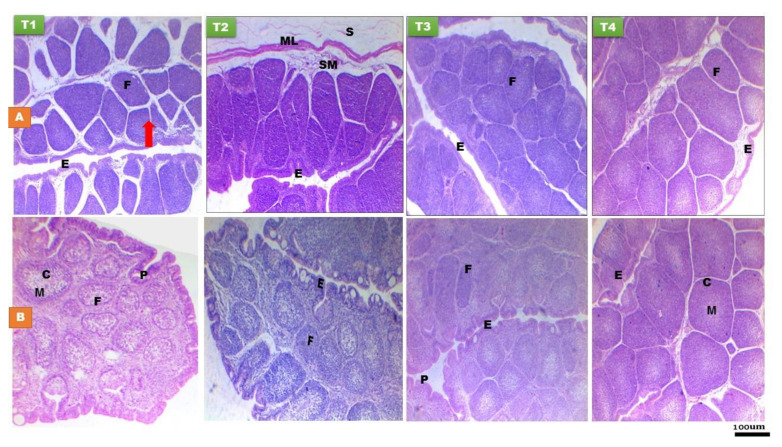
Histological structure of bursa of Fabricius at day 14 (**A**) and day 28 (**B**) of age of layer-type chicks exposed to fasting (control, T1) or early feeding on a layer starter diet (T2), a layer starter diet contained 3% molasses (T3), and a broiler starter diet (T4) during the first 72 h post-hatching. C, cortex; M, medulla; E, epithelium; P, Pelicae; F, follicle; SM, submucosa; ML, muscle layer; S, serosa. (H & E ×40).

Histomorphological characteristics of bursa of Fabricius at day 14 and 28 PH of layer-type chicks are presented in Figure 2 and Table 2 and Table 3. The histological structures of bursa were normal in all groups and at all time points (Figure 2). Bursa of Fabricius is consisted of folded mucosa lined with pseudostratified columnar epithelium. The mucosal folds are filled with numerous polyhedral follicles extended in lamina propria and the submucosa, which is separated by connective tissue, each follicle is composed of lymphatic tissue and divided into cortex and medulla. At day 28 PH, bursa is characterized by the presence of mucoid cysts in between the bursal epithelium (Figure 2). Greater relative weight of bursa (*p* < 0.01) was recorded in chicks fed the T2, T3, and T4 diets at day 14 of age compared to the fasted ones (Table 2). EFPH had significant effect (*p* < 0.01) on the Pelicae height and follicular width of bursa, characterized by higher values in the chicks fed on the T2, T3, and T4 diets at day 28 of age. Greater cortex thickness (*p* < 0.01) was recorded in the T2, T3, and T4 groups than in the fasted group at day 14 of age, while, it was only higher (*p* < 0.001) in the T4 group at day 28 of age than other treatments. There was non-significant difference in the bursal medulla among the treatment groups. The cortex to medulla ratio was higher (*p* < 0.01) in the chicks fed on the T4 diet than the other treatment groups at day 28 of age.

#### 3.1.3. Spleen

**Figure 3:**
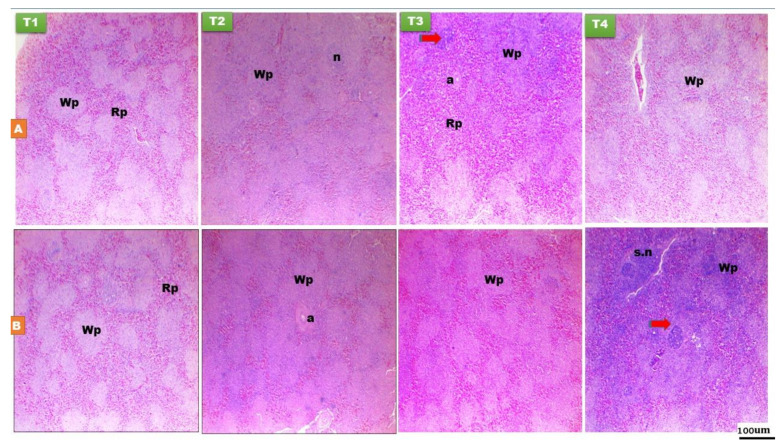
Histological structure of spleen at day 14 (**A**) and day 28 (**B**) of age of layer-type chicks exposed to fasting (control, T1) or early feeding on a layer starter diet (T2), a layer starter diet contained 3% molasses (T3), and a broiler starter diet (T4) during the first 72 h post-hatching. Histological examination showed variation in thickness of white pulp (Wp) and the red pulp (Rp), and germinal central area (red arrow). s.n, splenic nodules; n, nodule; a, central artery. (H and E ×40).

There was non-significant (*p* > 0.05) effect of EFPH on the splenic red pulp among the treatment groups (Figure 3 and Table 2 and Table 3). Splenic relative weight was greater (*p* < 0.05) in the T3 and T4 chicks at day 14 of age, while, there was non-significant difference in the splenic relative weight among the treatment groups at day 28 of age (Figure 3 and Table 2 and Table 3). Germinal center became noticeable on day 14 and 28 of age, nearby the arteries and typically obviously distinguished from the surrounding structures (Figure 3). Early nutrition PH increased the splenic white pulp and germinal center area in the early fed chicks at day 14 (*p* *<* 0.05) and 28 (*p* < 0.001) of age compared with the fasted group (Figure 3 and Table 2 and Table 3).

### 3.2. Liver and Proventriculus Histomorphology

#### 3.2.1. The Liver

**Figure 4:**
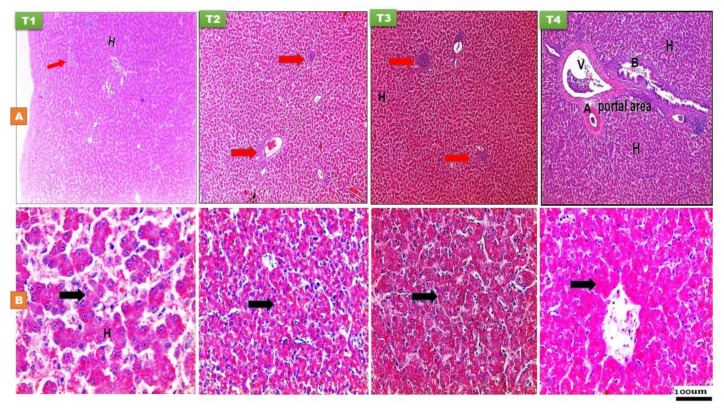
Histological structure of liver at day 14 (**A**) and day 28 (**B**) of age of layer-type chicks exposed to fasting (control, T1) or early feeding on a layer starter diet (T2), a layer starter diet contained 3% molasses (T3), and a broiler starter diet (T4) during the first 72 h post-hatching. Histological examination showed the arrangements of hepatocytes (H) with rounded basophilic nucleus, aggregation of lymph nodules in different area in parenchyma (red arrow) (H and E ×40). Portal area consisted of hepatic portal vein (V), hepatic artery (A), and bile duct (B). The distribution of glycogen granules appeared as reddish dots in the cytoplasm of hepatocytes (black arrow) using Best’s carmine stain (×100 and 200).

At 14 and 28 days PH, the hepatocytes appeared pyramidal in shape (Figure 4). The histomorphological examination of the liver was normal in all groups at both time points (Figure 4). The cytoplasm was more acidophilic without cytoplasmic vacuoles in addition to aggregations of lymphocytes were mostly aggregated in three regions in the liver such as around the portal area surrounding the blood vessels, around the central veins, and between the hepatic plates. The hepatocytes were arranged in two-cell-thick hepatic plates. The hepatic sinusoids were lined by flat endothelial cells and Von Kupffer cell. With Best’s carmine stain, Figure 4 revealed that the T2, T3, and T4 treatment groups had a strong positive reaction to the stain and significantly greater glycogen density score, both at day 14 and 28 of age, characterized by more reddish dots in the cytoplasm of hepatocytes, suggesting greater hepatic glycogen density (Figure 4).

#### 3.2.2. Proventriculus

**Figure 5:**
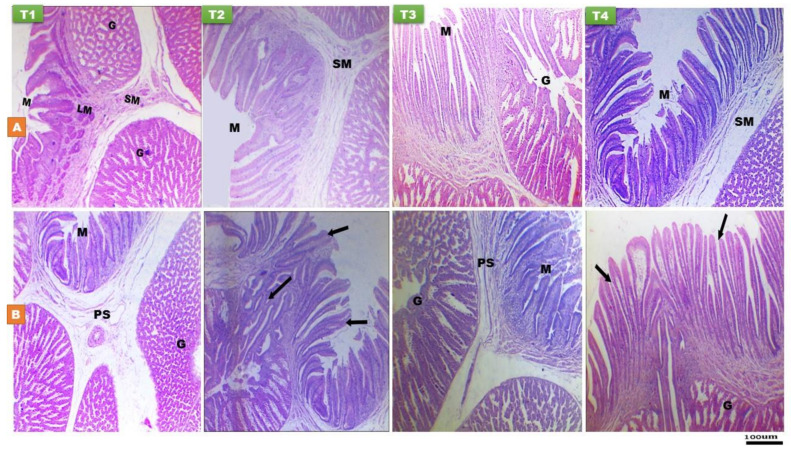
Histological structure of proventriculus at day 14 (**A**) and day 28 (**B**) of age of layer-type chicks exposed to fasting (control, T1) or early feeding on a layer starter diet (T2), a layer starter diet contained 3% molasses (T3), and a broiler starter diet (T4) during the first 72 h post-hatching. M, mucosa with longitudinal mucosal folds (black arrow); LM, lamina muscularis mucosae; PS, propria submucosa; SM, sub mucosa; G, submucosal gland. (H and E, ×40).

Histomorphological characteristics of proventriculus at day 14 and 28 PH of layer-type chicks are shown in Figure 5 and Table 2 and Table 3. The wall of proventriculus is consisted of mucosa which thrown into grossly folds, and it is composed of columnar cells while lobules of simple tubular gland consisted from cuboidal to low columnar secretory cells located in propria-submucosa, then surrounded by muscle and serosal layer (Figure 5). Greater relative weight of proventriculus was recorded in chicks fed the T3 and T4 diets compared to the FD ones (*p* < 0.05). The effect of early nutrition on the proventriculus histomorphology indicated that chicks fed the T2, T3, and T4 diet recorded the highest mucosal fold height at 14 day of age compared to fasted ones (*p* < 0.01), while the T4 chicks had the greatest mucosal fold height at day 28 of age (*p* < 0.001) compared to other treatments. At day 14 of age, there was a significant increase (*p* < 0.001) in the proventriculus mucosal thickness in the T3 and T4 groups compared to the other groups. A significant effect of early nutrition PH was observed on the proventriculus submucosal gland thickness, indicated higher values (*p* < 0.05) in the T2, T3, and T4 chicks compared to fasted ones at day 14 and 28 of age.

### 3.3. Immunity Related Gene Expression

**Figure 6:**
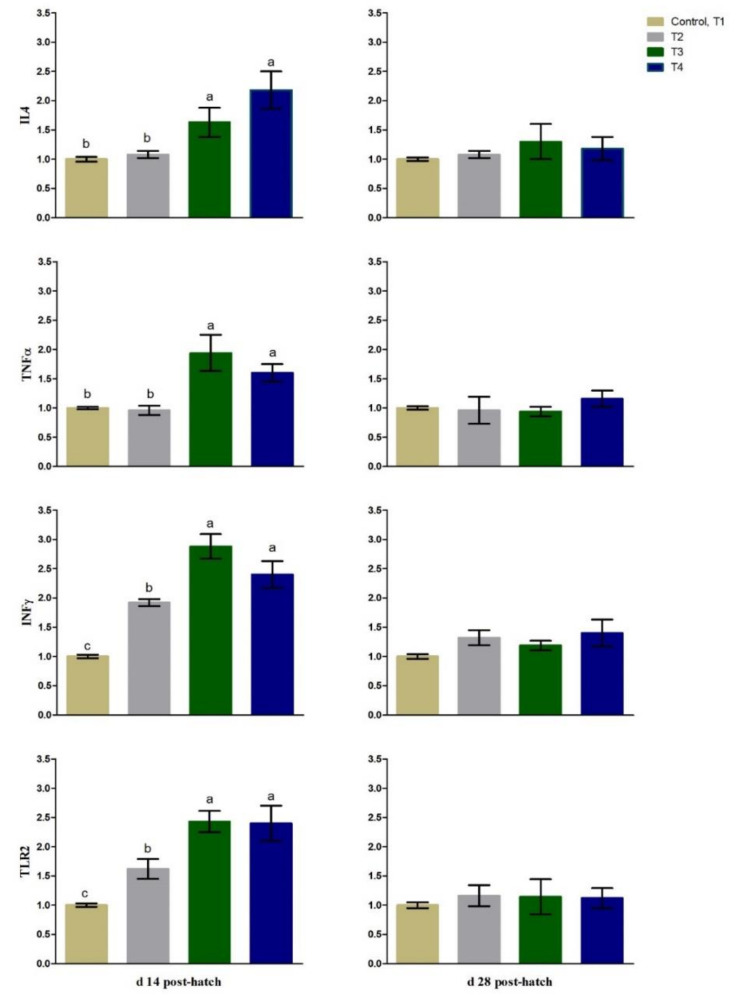
Splenic gene expression at day 14 and day 28 of age in layer-type chicks exposed to fasting (control, T1) or early feeding on a layer starter diet (T2), a layer starter diet contained 3% molasses (T3), and a broiler starter diet (T4) during the first 72 h post-hatching. IL4, interleukin 4; TNFα, tumor necrosis factor α; INFγ, interferon gamma; TLR2, Toll-like receptor 2. Expression of control is taken as 1.0. Data are presented as mean ± SE. ^a–c^ Means with different letters are significantly varied at *p* < 0.05.

The expression levels of splenic TLR2 and INFγ were up-regulated (*p* < 0.01) in most of the early fed chicks (T2, T3, and T4) compared to fasted ones (CON, T1) at 14 days PH (Figure 6). The mRNA expression of splenic IL4 and TNFα was up-regulated (*p* < 0.05) in the T3 and T4 groups compared to the T2 and fasted birds at day 14 of age (Figure 6). At 28 days PH, there was non-significant difference in the mRNA level of splenic TLR2, INFγ, IL4 or TNFα among the treatments (Figure 6). The expression levels at day 28 PH were non-significantly up-regulated (*p* > 0.05) in the fasted birds.

## 4. Discussion

Earlier studies on broilers [1,2,8,18] and layers [9,10] proposed that EFPH can influence their early immune system development, especially the relative lymphoid organs weights, and the immune response. However, to our knowledge little information is existing in the literature about the effect of EFPH or FD on the histomorphology of the liver, proventriculus, and lymphoid organs in layer-type chicks. Therefore, the current study aimed to determine the effect of EFPH versus FWDPH on the early development of the lymphoid (weak immune status) and digestive organs in layer-type chicks. This is because the immune organs are matured and fully developed (strong immune status) during the grower period of layers [9,28].

Thymus, bursa of Fabricius, and spleen are the main originators of the immune cells involved in the cellular and humoral immunity. The development and maturation of these organs appear more effectively in healthy birds than in diseased ones, and their growth can reveal the function and response of the immune system [1,2,6,12,25]. In the current study, EFPH increased the relative lymphoid organs weight, particularly the spleen and bursa of Fabricius, which is in agreement with previous studies in broiler- [2,6,8] and layer- [9] type chicks. In contrast, Shinde et al. [10] reported non-significant difference in the thymus, bursa, and spleen relative weights between the FD and early-fed layer-type chicks at 36 h, 7 day, and 14 day PH. Greater indexes of lymphoid organs in birds are commonly determined as indicators of the augmented proliferation of B and T lymphocytes, which implies better immunity [25]. Thymus is the main site of thymocytes, particularly T-lymphocytes, proliferation and maturation, bursa of Fabricius is essential for B-cell proliferation, and the spleen, the largest peripheral lymphoid organ, is included in the immune response [28,29]. The histomorphological structure of the thymus gland, in particular the cortex and medulla, can be affected by various stimulus [30]. The differences in the cortex and medulla have a linkage to the development, maturation, and function of the immune system [12,25,31]. In the current study, the histological structure of thymus was normal in all the experimental groups and at both time points, and in agreement with previous studies [12,25,31]. EFPH on the T3 and T4 diets increased the cortex of thymus, both at day 14 and 28 of age. In the cortex, there is an intensive proliferation and maturation of T lymphocytes, while the medulla is dominated by mature CD4+ or CD8+ cells [12]. The greater cortex in the T3 and T4 groups may suggest a faster proliferation and maturation of T-lymphocyte [32]. The noticeable increase in the cortex to medulla ratio in these early fed groups at day 28 of age revealed more mature CD4+ and CD8+ T cells migrate from the cortex to the medulla of thymus than in the fasted ones. These cells are known to recognize pathogens and can travel to the secondary lymphatic organs through the blood circulation [12]. It was reported that intensive development of thymus lobules was recorded between day 7 to day 21 PH [12]. Overall, these results suggested that EFPH with diets rich in protein and energy contents or contain easily available carbohydrates may modify the early development of the thymus in layer-type chicks. Madej et al. [12] reported that in ovo-injection of probiotic and/or synbiotic resulted in greater cortex and higher cortex to medulla ratio of thymus than non-injected group. To the best of our knowledge, no previous research is available in literature emphasizing the effect of EFPH on histomorphology of thymus to which we may compare our findings. It has been reported that the thymus gland is highly susceptible to FD, energy or amino acid deficiencies, which induce a rapid decrease in cellularity and weight [4,33]. Nutrient deprivation for long period may cause a reduction in the numbers of CD4+ and CD8+ T cells [4]. Furthermore, higher concentrations of CD4+ and CD8+ cells were observed in the early fed chicks compared to the FD ones [1].

The development of bursa occurs by the formation and colonization of the medulla during the embryonic stage, while the cortex develops after hatching [34]. Post-hatching, the B cells development takes place in the appearance of gut constituents such as antigens [35]. These antigens could possibly play a key role in the proliferation and/or differentiation of B cells [12,35]. The findings of the current trial showed that EFPH on the T2, T3, or T4 diets increased the bursa cortex and follicular width PH, suggesting an enhancement in the migration of B cells from the medulla to the cortex, which leading to acceleration in the follicular cortex development in the fed groups when compared with the fasted ones. The higher follicular width of the bursa suggested an increase in the B-lymphoblasts count, and as a result the formation of B-lymphocytes for antibodies [36]. Furthermore, EFPH could enhance the humoral immunity of chicks PH owing to the availability of nutrients essential to produce antibodies [36]. Juul-Madsen and Sorensen [1], Dibner et al. [2], and Bar Shira et al. [6] proposed that EFPH provides the birds with the essential nutrients, impacts the endogenous hormones or immunomodulators levels, and modifies the gut antigen repertoire, which in turn augments the differentiation of immune cells such as B lymphocytes. Supporting evidence was obtained by Simon et al. [9] who observed that EFPH resulted in an earlier onset of IgA expression in the early-fed (day 9 PH) compared with FD (day 14 PH) layer-type chicks. They suggested an antigenic stimulation through feed intake could be contributed to the B cell development [9]. Furthermore, the microbial sequence may have been postponed because of a delay in feed intake [9,20]. Another possible explanation was that fasting can stimulate the corticosteroids secretion and other metabolic changes, which in turn can diminish the proliferation of the immune organs [2]. 

The splenic index is directly correlated with the immune response [37]. The spleen has two main parts; red pulp and white pulp. The red pulp is mainly consisted of red blood cells, whereas white pulp is predominantly composed of lymphocytes. Histological structure of the spleen in the present trial was normal in all groups at both time points and was in accordance with the earlier studies [12,25]. In the spleen, the germinal center area is well-known as an essential histological structure of the spleen stimulation [12,25]. It was recorded that the splenic germinal center area was noticed with clear histomorphological structure at day 14, 21, and 35 of age [11,12]. In the current study, an increased white pulp and germinal center area of the spleen along with a greater spleen index in the early fed groups when compared with the fasted chicks, suggesting that EFPH may have stimulated the splenic B cells proliferation, which in turn augmented the immune response [12,25]. In support of this, EFPH, particularly the T3 and T4 groups, up-regulated the splenic gene expression of IL4, TLR2, TNFα, and INFγ, which could enhance B cells proliferation and differentiation. The B and T lymphocytes have a key role in the immune response into which native T cells differentiate into Th1 cells (cellular immunity) and Th2 cells (humoral immunity) [21]. It has been recorded that glucose is an important source of energy for Th2 cells proliferation [38] and was observed to be decreased in fed deprived chicks for 24 h or more PH [39]. Shinde et al. [10] reported that the humoral and cellular immune responses was considerably improved in the FD layer-type chicks for 6, 12, and 24 h than those FD for 36 h PH. Moreover, Bakyaraj et al. [40] and Bhanja et al. [41] found that in ovo injection of amino acid and glucose enhanced the cellular and humoral immunity, as well as up-regulate the IL4, TLR2, TNFα, INFγ, and TNFα genes in chicks during the PH life, indicating their role in the development of the immune system of chicks. In agreement with our findings, Tamboli et al. [8] reported down-regulation of the splenic IL6, INFγ, TNFα, and TLR2 in the fasted birds compared to the early fed ones. Moreover, toll-like receptors (TLRs) was known to have a key role in innate immunity. Following the infection, TLRs initiate a signaling cascade for the cytokines production and up-regulate the co-stimulatory molecules [42]. In the current trial, the splenic TLR2 expression was up-regulated in the early fed chicks PH during the early stage of life. On contrary, Shinde et al. [10] observed up-regulation of splenic cytokines (TLR2 and IL6) as the FD period increased in layer chicks, and they did not observe any connection between in vivo immune response and immunity-related gene expression.

In birds, the liver has a wide range of metabolic and homeostatic functions [43]. At day 14 and 28 PH, the histological examination of the liver revealed the presence of lymphatic cell aggregations that are variable in number and size, and with an uneven scattering in the parenchyma. It has been reported that the hepatic lymphatic aggregations in birds are considered as a part of the peripheral lymphoid tissue that is a key constituent of the lymphatic system due to birds do not have distinctive lymph nodes [44,45]. Lymphoid tissue was recognized in non-immune organs in birds, including liver, pancreas, kidney, endocrine glands, gonads, and the central nervous tissue [43,44,45]. It is still unknown that are these lymphoid tissues a burst of lymphomatosis or a normal reaction of the avian immune system to antigens [46]. Little is known about the liver of layer-type chicks, particularly under the influence of early nutrition or FDPH and thus this point needs to be further explained in future studies.

The liver had an intense positive reaction to Best’s carmine stain and a greater glycogen density score in the early fed groups of this study, indicating that the liver of these chicks had numerous glycogen granules in the cytoplasm of hepatocytes. The liver glycogen concentration (majority) and muscle (minority) varies during the embryonic stage [47]. One day PH, both the heart and liver glycogen contents decline markedly to 40 and 16% of pre-hatching levels, respectively [48]. The glycogen contents in the liver persist at a lower level for several weeks PH and rise to the adult concentrations at 4 months of age [47]. Hepatic glycogen is constantly being formed and break down, its content varied depending on the carbohydrate (gluconeogenic substrates) intake and the glycogenolysis [47]. In the current study, EFPH or FWDPH may influence the carbohydrate metabolism of the liver in layer-type chicks, however the exact mechanism of action was not investigated. In line with the current findings, Kornasio et al. [49] observed that administration of carbohydrate before hatch via in ovo feeding combined with EFPH maximized the carbohydrate availability and elevated liver glycogen content. Furthermore, the early fed chicks had more than 30-fold increment in the hepatic glycogen content compared to the FD chicks [49].

The proventriculus is the glandular stomach where hydrochloric acid and digestive enzymes are produced for the digestion process of feeds. In the present study, the histomorphological structure of proventriculus was normal and coincide with previous studies [26,50,51]. Our data revealed that the mucosal fold thickness, proventriculus mucosal thickness, and diameter of compound tubular gland lumen were greater in the fed groups PH than the fasted group. It has been reported that the relative weight of proventriculus increased gradually from day 1 to day 28 of age in birds [52]. Increased thickness of the proventriculus mucosa and gland are important indicators of the proventriculus development and suggesting that early feeding PH increased the production of the gastric juice, which is essential for digestion [53]. This effect has been accompanied by a rapid growth rate of the studied chicks as was reported in the previously published data of our study [7]. Previous researches have recorded the impacts of PHFD on the relative digestive organs’ weights of chickens, including the liver, proventriculus, gizzard, intestinal weight, and length [7,54]. It can be assumed that PHFWD delays the digestive organs development, particularly the proventriculus and the liver. In support of this, Maiorka et al. [55] observed that the FD chicks had lower relative proventriculus and gizzard weights after 48 h and 72 h of fasting compared to the fed ones.

## 5. Conclusions

The findings of this study reveal that EFPH with the experimental diets had no negative effect on the development of the immune and digestive organs. FWDPH reduced the cortex and cortex:medulla in the thymus and dampened the growth of the cortex, follicular width, and Pelicae height in bursa of Fabricius, with resultant influences on the primary lymphoid organs. Early nutrition PH enhanced the formation of germinal center areas in the spleen of early fed chicks, implying augmented proliferation of B cells in the secondary lymphoid organs. Early nutrition PH, particularly with diets rich in protein and energy contents or contain easily digestible carbohydrate, had an intense positive reaction to Best’s carmine stain in the liver of these groups, indicating that the liver of these chicks had numerous glycogen granules in the cytoplasm of hepatocytes. Improved proventriculus mucosal and gland thickness, as well as fold height were observed in the early fed chicks at day 14 of age, suggesting an increase in the production of the gastric juice, which is essential for digestion. The expression levels of splenic TLR2, IL4, TNFα, and INFγ were up-regulated in most of the early fed chicks compared to fasted ones at day 14 of age. In conclusion, EFPH could modify the splenic-immunity related genes and modulate the histomorphology of the lymphoid and digestive organs (liver and proventriculus) in layer-type chicks during their early life PH.

## Figures and Tables

**Table 1:** Oligonucleotide sequence of splenic gene primers.

Gene ^1^	Sequence (5′-3′)	Annealing Temperature (°C)	Product Size (bp)	Accession Number	Reference
IL4	F-AATGACATCCAGGGAGAGGTTTC R-GCTAGTTGGTGGAAGAAGGTACG	55	219	JN639847	[3]
TNFα	F-AGACCAGATGGGAAGGGAATGAA R-GAAGAGGCCACCACACGACAG	55	219	JN942589	[8]
INFγ	F-AGCTGACGGTGGACCTATTATTGT R-CGGCTTTGCGCTGGATTC	58	260	JN942588	[3]
TLR2	F-GTGGCCATGTCGATCAGCAGAAAC R-TCAGCGGAGAGTCACAGATGTAGC	56	202	NM_204278 ^1^	[8]
28S rRNA	F-CAGGTGCAGATCTTGGTGGTAGTA R- GCTCCCGCTGGCTTCTCC	58	273	JN639848	[3]

^1^ IL4, interleukin 4; TNFα, tumor necrosis factor alpha; INFγ, interferon gamma; TLR2, Toll-like receptor 2.

**Table 2:** Effect of early nutrition with different diets post-hatching (PH) on the relative weights (% of live body weight) and histomorphology of lymphoid organs and proventriculus (µm) of layer-type chicks at day 14 of age.

Items ^2^	Treatments ^1^				SEM	*p*-Value
	CON-T1	T2	T3	T4		
***Lymphoid organs***						
***Thymus gland***						
Relative weight	0.215	0.241	0.253	0.243	0.011	0.11
Cortex	263.31 ^b^	263.97 ^b^	348.38 ^a^	258.95 ^b^	38.70	0.03
Medulla	182.19	184.26	218.07	218.07	12.85	0.13
Cortex:Medulla	1.45	1.43	1.60	1.19	0.142	0.11
***Bursa of Fabricius***						
Relative weight	0.340 ^b^	0.415 ^a^	0.440 ^a^	0.495 ^a^	0.018	0.003
Pelicae height	23.37 ^c^	57.70 ^b,c^	88.87 ^b^	144.40 ^a^	12.57	<0.001
Follicle width	123.63	172.01	191.56	174.22	22.53	0.08
Cortex	232.18 ^b^	397.96 ^a^	377.40 ^a^	298.00 ^a,b^	32.55	0.003
Medulla	133.28	171.13	148.71	144.61	16.28	0.21
Cortex:Medulla	1.74	2.35	2.55	2.13	0.309	0.13
***Spleen***						
Relative weight	0.125 ^b^	0.130 ^b^	0.145 ^a^	0.140 ^a^	0.003	0.04
Red pulp	145.27	173.23	132.89	158.64	12.88	0.09
White pulp	141.30 ^b^	161.44 ^b^	154.11 ^b^	223.59 ^a^	25.17	0.03
GCA	0.21 ^b^	0.40 ^a,b^	0.50 ^a^	0.52 ^a^	0.082	0.04
***Digestive organs***						
***Proventriculus***						
Relative weight	0.950 ^b^	1.05 ^a,b^	1.180 ^a^	1.170 ^a^	0.032	0.01
Fold height	303.56 ^b^	700.76 ^a^	565.50 ^a^	580.69 ^a^	71.39	0.003
Mucosa	496.22 ^b^	600.92 ^b^	1035.28 ^a^	1210.45 ^a^	89.09	<0.001
Gland	695.54 ^b^	886.75 ^a^	834.37 ^a^	829.36 ^a^	64.19	0.04
Lumen	94.46 ^b^	218.51 ^a^	186.91 ^a^	126.33 ^b^	17.87	<0.001
***Liver***						
Glycogen density	1.42 ^c^	2.41 ^b^	2.75 ^b^	3.42 ^a^	0.288	0.001

^a–c^ values in the same row with different letters are significantly varied at *p* ˂ 0.05, SEM, pooled standard error of the means. ^1^ CON-T1, no feed or water during the first 72 h of PH; T2, chicks fed on a layer starter diet contained 20% CP and 11.84 MJ/kg; T3, chicks fed on a layer starter diet contained 20% CP and 11.81 MJ/kg (diet contained 3% molasses) and T4, chicks fed on a broiler starter diet contained 23% CP and 12.68 MJ/kg during the first 72 h PH. The experimental diets were provided on *ad libitum*. All birds after 72 h PH were fed a commercial layer starter mash diet (the diet T2) ad libitum for 42 days ^2^. GCA, germinal center area.

**Table 3:** Effect of early nutrition with different diets post-hatching (PH) on the relative weights (% of live body weight) and histomorphology of lymphoid organs and proventriculus (µm) of layer-type chicks at day 28 of age.

Items ^2^	Treatments ^1^				SEM	*p*-Value
	CON-T1	T2	T3	T4		
***Lymphoid organs***						
***Thymus gland***						
Relative weight	0.230	0.250	0.265	0.260	0.013	0.33
Cortex	278.20 ^b^	353.38 ^a^	396.71 ^a^	408.38 ^a^	31.13	0.01
Medulla	217.23	227.10	226.25	209.74	35.48	0.98
Cortex:Medulla	1.28 ^b^	1.55 ^a,b^	1.75 ^a^	1.95 ^a^	0.11	0.03
*Bursa of Fabricius*						
Relative weight	0.240	0.253	0.250	0.251	0.016	0.36
Pelicae height	99.20 ^c^	135.39 ^b^	169.81 ^a^	161.33 ^a^	15.76	0.008
Follicle width	189.71 ^c^	163.78 ^c^	233.15 ^b^	314.91 ^a^	21.06	<0.001
Cortex	212.67 ^b^	223.21 ^b^	246.24 ^b^	521.65 ^a^	37.68	<0.001
Medulla	167.12	137.83	159.93	147.84	16.69	0.37
Cortex:Medulla	1.30 ^b^	1.62 ^b^	1.56 ^b^	3.65 ^a^	0.462	0.003
*Spleen*						
Relative weight	0.115	0.120	0.125	0.135	0.009	0.28
Red pulp	179.41	165.20	181.99	169.73	19.13	0.79
White pulp	126.51 ^c^	180.41 ^b^	163.61 ^b^	231.20 ^a^	20.35	0.009
GCA	0.37 ^c^	0.55 ^b^	0.66 ^a^	0.63 ^a,b^	0.038	<0.001
***Digestive organs***						
***Proventriculus***						
Relative weight	0.895	0.935	1.015	0.965	0.048	0.17
Fold height	493.88 ^b^	584.23 ^b^	597.53 ^b^	1182.03 ^a^	59.41	<0.001
Mucosa	973.42	953.84	944.59	1118.86	125.54	0.34
Gland	624.79 ^b^	892.82 ^a^	1039.14 ^a^	855.09 ^a^	84.93	0.008
Lumen	179.03	271.63	288.64	261.50	33.07	0.20
*Liver*						
Glycogen density	1.75 ^c^	2.43 ^b,c^	3.08 ^a,b^	3.50 ^a^	0.317	0.003

^a–c^ values in the same row with different letters are significantly varied at *p* ˂ 0.05, SEM, pooled standard error of the means. ^1^ CON-T1, no feed or water during the first 72 h of PH; T2, chicks fed on a layer starter diet contained 20% CP and 11.84 MJ/kg; T3, chicks fed on a layer starter diet contained 20% CP and 11.81 MJ/kg (diet contained 3% molasses) and T4, chicks fed on a broiler starter diet contained 23% CP and 12.68 MJ/kg during the first 72 h PH. The experimental diets were provided on *ad libitum*. All birds after 72 h PH were fed a commercial layer starter mash diet (the diet T2) *ad libitum* for 42 days ^2^. GCA, germinal center area.

## Data Availability

The data presented in this study are available on request from the corresponding author.
